# Towards next generation therapies for cystic fibrosis: Folding, function and pharmacology of CFTR^☆^

**DOI:** 10.1016/j.jcf.2019.12.009

**Published:** 2020-03

**Authors:** Samuel J. Bose, Georg Krainer, Demi R.S. Ng, Mathias Schenkel, Hideki Shishido, Jae Seok Yoon, Peter M. Haggie, Michael Schlierf, David N. Sheppard, William R. Skach

**Affiliations:** a School of Physiology, Pharmacology and Neuroscience, University of Bristol, Bristol BS8 1TD, UK; b B CUBE – Center for Molecular Bioengineering, Technische Universität Dresden, Tatzberg 41, 01307 Dresden, Germany; c CFFT Lab, Cystic Fibrosis Foundation, Lexington, MA 02421, USA; d Department of Medicine, University of California, San Francisco, CA 94143-0521, USA; e Cystic Fibrosis Foundation, Bethesda, MD 20814, USA

**Keywords:** CFTR Cl^−^ channel, F508del-CFTR, Rare CF mutations, Protein folding, CFTR correction, CFTR potentiation

## Abstract

The treatment of cystic fibrosis (CF) has been transformed by orally-bioavailable small molecule modulators of the cystic fibrosis transmembrane conductance regulator (CFTR), which restore function to CF mutants. However, CFTR modulators are not available to all people with CF and better modulators are required to prevent disease progression. Here, we review selectively recent advances in CFTR folding, function and pharmacology. We highlight ensemble and single-molecule studies of CFTR folding, which provide new insight into CFTR assembly, its perturbation by CF mutations and rescue by CFTR modulators. We discuss species-dependent differences in the action of the F508del-CFTR mutation on CFTR expression, stability and function, which might influence pharmacological studies of CFTR modulators in CF animal models. Finally, we illuminate the identification of combinations of two CFTR potentiators (termed co-potentiators), which restore therapeutically-relevant levels of CFTR activity to rare CF mutations. Thus, mechanistic studies of CFTR folding, function and pharmacology inform the development of highly effective CFTR modulators.

## Highlights

• Co-translational folding of NBD1 is disrupted by CF mutations.
• FRET analysis of transmembrane constructs reveals misfolding by CF mutations.
• The impact of the F508del mutation on CFTR is species-dependent.
• Two potentiators with distinct actions restore CFTR activity to rare CF mutations.

## 1 Introduction

Thirty years after the identification and cloning of the cystic fibrosis transmembrane conductance regulator (CFTR) [1], a transformational drug therapy for most people with cystic fibrosis (CF) received regulatory approval [2,3]. The development of Trikafta™ (elexacaftor-tezacaftor-ivacaftor) (Vertex Pharmaceuticals) is the culmination of great efforts to understand the structure of CFTR, its synthesis in epithelial cells, function as a ligand-gated anion channel and modulation by small molecules [4]. Despite the enormous progress, CFTR modulators are not yet available to all people with CF, while there is evidence of disease progression in CFTR modulator-treated patients (for review, see [4]). Thus, there remains a pressing need to understand even better CFTR, its biosynthesis in cells, structure-function relationships and physiological roles. This knowledge will inform the development of next generation small molecule CFTR modulators, leading to life-long personalised treatments for CF.

Here, we review selectively some recent technical innovations to understand better how CF mutations perturb the assembly of CFTR, highlight species-dependent differences in CFTR expression, stability and function relevant to CF animal models and identify combinations of two small molecule CFTR potentiators that restore function to rare CF mutations, which have so far proven unresponsive to CFTR modulators. This review is based on symposia presentations from the 16th European Cystic Fibrosis Society Basic Science Conference, Dubrovnik, Croatia, 27–30th March 2019. Although CFTR folding, function and pharmacology are broad topics that each merit their own review, here they are combined to highlight the importance of mechanistic insight for CF drug discovery and development. For more comprehensive reviews of CFTR folding, function and pharmacology, we refer the Reader to [5], [6], [7], [8], [9].

## 2 CFTR folding: mechanism of CFTR cotranslational folding

**Fig. 1:**
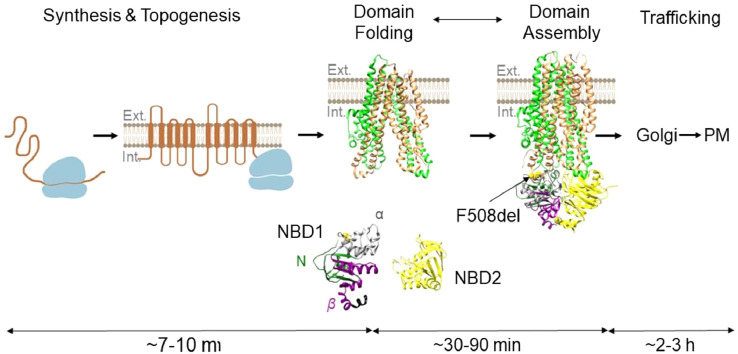
CFTR folding in the cell. The schematic shows the time scale of CFTR synthesis, folding and domain assembly in cells. The sub-domains of NBD1 (N-terminus (N), K377 – S492; α-helical (α), Q493 – D565; α/β-core (β), A566 – Q637) and the position of the F508del mutation are indicated. *Abbreviations:* NBD, nucleotide-binding domain; PM, plasma membrane. The figure was created, in part, with BioRender.com using CFTR structures (PDB id: 5UAK and 6MSM) and therefore lacks the unstructured R domain.

Cotranslational folding is a highly dynamic process that takes place as the nascent polypeptide is progressively synthesized from N- to C-terminus in a complex biological environment. This process is influenced by the adjacent ribosome [10,11], the translation rate [12,13], cellular chaperones [14,15], and the cytosolic folding environment [16]. For CFTR, cotranslational folding requires the precise insertion of 12 transmembrane α-helices into the lipid bilayer, tertiary folding and packing of three cytosolic domains and two membrane-spanning domains (MSDs), and assembly of these domains into a mature tertiary structure (Fig. 1). Many of these steps occur during the 7–10 minutes required to complete CFTR peptide synthesis and thus are truly cotranslational events [17]. The final steps of post-translational folding, including domain packing and assembly, are likely orchestrated after synthesis by cytosolic folding machinery prior to exit from the endoplasmic reticulum and delivery to the plasma membrane and take 30–90 min [17].

CFTR maturation relies crucially on proper folding of individual domains, and inherited mutations that disrupt any steps in this process lead to a misfolded protein that is degraded by the ubiquitin proteasome pathway (UPP) via a process known as Endoplasmic Reticulum Associated Degradation (ERAD) [5,17]. Although, the first nucleotide-binding domain (NBD1) is a particular hot-spot for trafficking mutations and plays a major role in overall CFTR folding efficiency [18], [19], [20], it is unknown when and how such mutations exert their effect. To determine whether trafficking mutations influence NBD1 folding cotranslationally or posttranslationally, Skach and coworkers [11], [21] developed an assay using Fluorescence (Förster) Resonance Energy Transfer (FRET) between two fluorophores that were cotranslationally incorporated into the growing nascent polypeptide to identify the stage of synthesis at which specific compaction events occur.

Using this FRET assay, Khushoo et al. [21] and Kim et al. [11] showed that NBD1 folds cotranslationally during synthesis on the ribosome via the sequential compaction of N-terminal, α-helical, and α/β-core subdomains (Fig. 1). The timing of these folding events is finely tuned by properties of the ribosome that delay collapse of the α-subdomain until synthesis of a 4-stranded parallel β-sheet core is completed. For example, premature release of the polypeptide from the ribosome results in rapid folding of the α-subdomain and irreversible failure of the core β-sheet to form. Because the timing of these folding events is critical, Kim et al. [11] tested whether the rate of translation influences coupling of the α-subdomain and β-core folding by introducing synonymous codon changes that were predicted to increase the translation rate precisely when the α-subdomain exited the ribosome (CFTR residues 525–593, “Fast-CFTR”), while keeping the amino acid sequence unchanged. These synonymous changes had little effect on CFTR synthesis or processing efficiency. However, they altered CFTR biogenesis in such a way as to induce a delayed aggregation of NBD1 and hence, full-length immature CFTR protein. Moreover, these synonymous codon changes also induced structural changes in epitopes on NBD1 and full-length CFTR that were related to the rate of cotranslational folding and were independent of CFTR sequence. Thus, an altered local epitope conformation within the native peptide sequence was cotranslationally imprinted and preserved throughout CFTR processing and intracellular trafficking [11]. Indirect analysis of the translation rate by ribosome profiling further confirmed ribosomal pausing within the region of synonymous codon changes that was abolished in the “Fast-CFTR” construct, indicating that translation rate can impact the efficiency of the overall folding outcome. Consistent with these data, *in silico* analyses of synonymous single nucleotide polymorphisms (sSNPs) identified sSNPs which have the potential to change CFTR structure, so called “non-silent” synonymous mutations [22] (see also Kirchner et al. [23]). Taken together, the data suggest that restoration of cotranslational folding dynamics might provide an important therapeutic strategy for CF and other folding disorders.

## 3 CFTR folding: analysis of transmembrane helices with single-molecule FRET

CF mutations located in the MSDs frequently cause misfolding (e.g. [24,25]). Analysis of their effects on CFTR folding are mostly investigated indirectly by evaluating protein maturation rates in cells. However, such analyses preclude insight into how CF mutations cause misfolding of transmembrane helices and how CFTR correctors reverse misfolding. A significant challenge in the development of such assays is the inherent complexity of studying the folding of full-length CFTR protein. The full-length protein with its 1,480 amino acids is notoriously difficult to obtain in sufficient quantities and purity for *in vitro* analysis, a problem confounded by CF mutations, which destabilise CFTR. Efforts to overcome this lack of protein stability recently culminated in a functional CFTR construct with six stabilising mutations in NBD1 [26]. Nevertheless, effective characterisation of the contribution of individual CF mutations to CFTR misfolding remains a challenge using classical biochemical and biophysical techniques due to the large size of the CFTR protein [27]. Such techniques are often limited in their ability to resolve the structural heterogeneities of misfolded protein states.

**Fig. 2:**
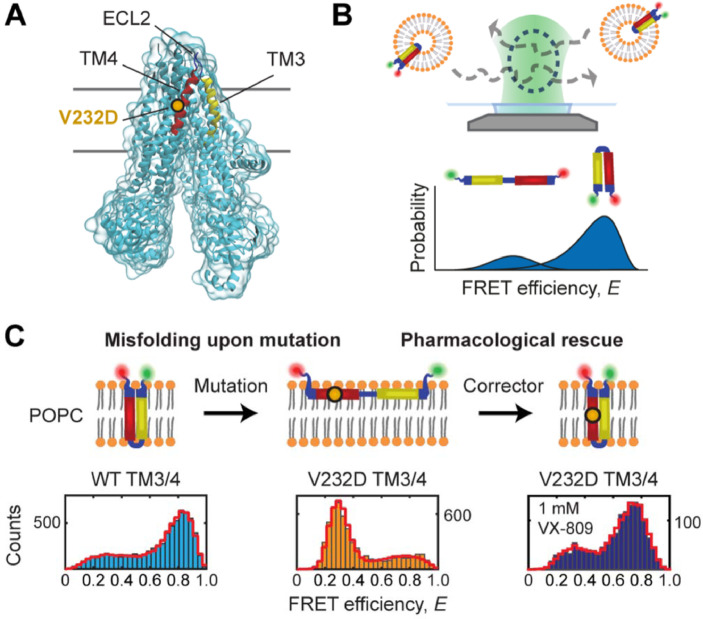
A minimal helical-hairpin motif provides molecular-level insights into misfolding and pharmacological rescue of CFTR. (A) Structure of CFTR (PDB id: 5UAK) highlighting the position of the V232D mutation in TM3/4 (yellow/red) and the intervening second extracellular loop (ECL2). (B) Schematic of the single-molecule FRET approach for investigating hairpin conformations. Shown are single fluorescently labeled TM3/4 hairpin molecules reconstituted into phospholipid vesicles freely diffusing through the observation volume of the confocal microscope. Obtained FRET efficiency histograms report on coexisting conformational hairpin states and their relative occupancies. (C) FRET efficiency histograms of wild-type (WT) TM3/4 (light blue), V232D TM3/4 (orange) and V232D TM3/4 in the presence of lumacaftor (VX-809; dark blue). Figure adapted from Krainer and Treff et al. [28] under Creative Commons Attribution License. (For interpretation of the references to colour in this figure legend, the Reader is referred to the web version of this article).

To gain molecular-level insights into CFTR misfolding and drug rescue of misfolded states, Krainer and Treff et al. [28] developed a single-molecule FRET-based approach that exploits helical-hairpin constructs derived from full-length CFTR as minimalist *in vitro* systems (Fig. 2A and B). Helical hairpins, comprising two transmembrane (TM) helices and their intervening loop region, are readily prepared in sufficient amounts for biophysical analysis [29,30]. They represent the smallest units that can be inserted autonomously by the translocon since CFTR topogenesis in the ER is based on pairwise integration of helical segments [17]. In tandem with single-molecule FRET [31,32], which serves as a spectroscopic ruler to probe the end-to-end distances of hairpins reconstituted in lipid bilayers (Fig. 2B), these minimalist folding units constitute versatile platforms to characterise biophysically the molecular events of CFTR folding.

Krainer and Treff et al. [28] applied this hairpin approach to study misfolding of the CF mutation V232D in TM4 (Fig. 2A) and the impact of the clinically-approved CFTR corrector lumacaftor [33] by exploiting a helix/loop/helix hairpin construct [34] comprising TM3 and TM4 of CFTR. The folding of TM3/4 was tracked by monitoring the FRET efficiency of single-molecule hairpin molecules reconstituted in lipid-bilayer membranes (Fig. 2C). When compared with the wild-type hairpin, the TM3/4 V232D mutant hairpin was misfolded in lipid membranes resembling the thickness of the endoplasmic reticulum, assuming an open structure associated with the interfacial region of the membrane [28]. These data suggest a model for V232D pathogenesis whereby partitioning of the TM3/4 V232D into the interfacial region traps CFTR as a partially folded intermediate at the membrane (Fig. 2C). They also rationalise cell-based analyses of CFTR misprocessing by the V232D mutation [35], arguing that the minimal hairpin motif might be used as a proxy to study the *in vivo*/*in vitro* correlates of CFTR misfolding.

Using the TM3/4 hairpin, Krainer and Treff et al. [28] investigated the action of lumacaftor on the CF mutation V232D [35,36]. Strikingly, lumacaftor reversed the open hairpin structure of TM3/4 V232D to restore a compact structure similar to that of the wild-type hairpin (Fig. 2C). This result suggests that the action of lumacaftor stabilises the native or near-native state of CFTR. In summary, this minimal *in vitro* approach constitutes a versatile platform to characterise the molecular events that link CF to structural effects of mutations, opening new avenues for the development of mechanism-based therapeutics that target CFTR and other misfolding-prone helical membrane proteins.

## 4 CFTR function: species-dependent differences inform CF mutation studies

Comparative studies of CFTR orthologues have demonstrated that CFTR misfolding is highly species dependent and identified structural motifs important for CFTR processing and plasma membrane stability [37,38]. Following identification of the *CFTR* gene [1], CFTR orthologues have been found in many jawed vertebrates (for CFTR phylogenic relationships, see [39]) and recently in the jawless vertebrate lamprey [40]. To understand better the pathogenesis of CF and evaluate new therapeutics for the disease, CF animal models have been developed using zebrafish, mice, rats, ferrets, pigs, sheep and rabbits [41], [42], [43], [44], [45], [46], [47]. By contrast, understanding of the single-channel behaviour of CFTR orthologues from these species and the impact of CF mutations upon them is incomplete.

**Fig. 3:**
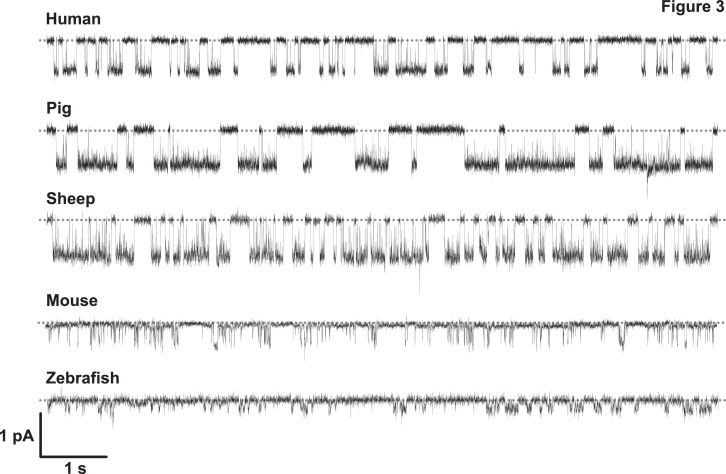
The single-channel behaviour of CFTR orthologues. Representative single-channel recordings of human, pig, sheep, mouse and zebrafish CFTR in excised inside-out membrane patches from Chinese hamster ovary (CHO) cells expressing the indicated CFTR variants or a C127 cell expressing human CFTR. The recordings were acquired using the experimental conditions described in Cai et al. [57] except that voltage was –100 mV for zebrafish CFTR. Dotted lines indicate where channels are closed and downward deflections correspond to changes in current following channel opening. Note that the sub-conductance state of mouse CFTR is not apparent without further filtering of single-channel records [54,58]. Figure adapted in part from Cai et al. [57] and Bose et al. [58] under Creative Commons Attribution License.

Comparison of the first cryo-EM structures of CFTR revealed high degrees of structural similarity despite only 54% amino acid identity between human and zebrafish CFTR [48], [49], [50], [51]. Thus, the striking differences in function between human and zebrafish CFTR were unexpected and not accounted for by codon optimisation [6, 52]. These differences include, first, zebrafish CFTR has an inwardly rectifying current–voltage (I–V) relationship unlike the ohmic I–V relationship of human CFTR [52]. Second, the gating pattern of zebrafish CFTR is dominated by prolonged channel closures with only brief channel openings (Fig. 3), despite similar ATP dependence to human CFTR [52]. Third, zebrafish CFTR exhibits marked differences in pharmacology, including the failure of ATP analogues to enhance channel activity and altered sensitivity to channel inhibitors [52]. Interestingly, the single-channel behaviour of zebrafish CFTR shows some similarities to mouse CFTR, the mammalian orthologue with greatest amino acid sequence difference from human CFTR (Fig. 3) [52], [53], [54].

Ostedgaard et al. [37] first demonstrated that the impact of the F508del mutation on CFTR is species dependent. Like human F508del-CFTR, ferret and sheep F508del-CFTR fail to mature, whereas mouse, pig, rabbit and especially chicken F508del-CFTR produce substantially more mature CFTR protein [38,55,56]. Less is known about the species dependence of F508del-CFTR thermostability, which is potentially confounded by cell type-dependent differences in the expression of regulatory molecules, such as protein phosphatases. However, the available data suggest a gradation of severity with human F508del-CFTR exhibiting marked thermoinstability, sheep intermediate and chicken and mouse F508del-CFTR little or none [38,57,58]. Interestingly, in all species studied, the severity of the F508del-CFTR gating defect is noticeably reduced when compared with that of human F508del-CFTR [37,38,55,57,58]. Taken together, the data suggest that subtle structural differences between CFTR homologues, including the I539T revertant mutation [59] and proline residues at dynamic locations in NBD1 [38], account for the spectrum of effects of the F508del mutation on CFTR processing, thermostability and channel gating.

Using a CF ferret with the G551D mutation, Sun et al. [60] demonstrated that *in utero* and postnatal administration of the clinically-approved CFTR potentiator ivacaftor [61] rescues disease progression. However, species dependent differences in pharmacology caution that small molecule CFTR modulators should be tested on CFTR orthologues expressed in heterologous cells before they are used in CF animal models. For example, fifteen CFTR correctors, including lumacaftor, rescued both human and mouse F508del-CFTR [58]. By contrast, twelve CFTR potentiators had differential effects on human and mouse F508del-CFTR with eight, including ivacaftor, enhancing human, but not mouse F508del-CFTR activity and one compound, genistein, potentiating human and mouse F508del-CFTR by distinct mechanisms [58].

A potential explanation for the different effects of ivacaftor on human and mouse F508del-CFTR is provided by the identification of a binding site for ivacaftor and a second potentiator GLPG1837 [62,63]. The tyrosine residue at position 304 in TM5 of human CFTR, which forms a hydrogen bond with the bound potentiator, is replaced by a phenylalanine residue in mouse CFTR [62,63]. Transfer of this tyrosine residue to mouse CFTR (i.e. mouse F304Y-CFTR), confers potentiation by ivacaftor and GLPG1837 on mouse CFTR [63]. However, the different effects of ivacaftor on mouse models of autoimmune disease [64] might require a different explanation. One possibility is the action of ivacaftor on the solute carriers (SLCs) SLC6A14, SLC26A3 and SLC26A9, which modify CFTR function and hence, the severity of CF [65]. Alternatively, acute and chronic ivacaftor might have different effects on mouse CFTR [58,64,66]. Future studies should address these possibilities.

## 5 CFTR pharmacology: development of novel therapeutic paradigms for rare CF mutations

Despite advances in understanding how CF mutations alter CFTR folding and function and the development of robust model systems for modulator evaluation, there remains an unmet need for new therapeutics. Small molecule CFTR modulators remain the only viable therapeutic approach to correct the ion transport defect in CF epithelia. To date, four CFTR modulator therapies, all developed by Vertex Pharmaceuticals, are in clinical use: ivacaftor (Kalydeco^Ⓡ^) for G551D-CFTR and 37 additional gating mutations [67], [68], [69]; lumacaftor-ivacaftor combination therapy (Orkambi^Ⓡ^) for F508del-CFTR homozygous subjects [70], and tezacaftor-ivacaftor combination therapy (Symdeko^Ⓡ^/Symkevi^Ⓡ^) for homozygous F508del-CFTR subjects, or subjects with F508del-CFTR and a residual function allele [71,72]. The latest combination therapy to receive regulatory approval (Trikafta™ (elexacaftor-tezacaftor-ivacaftor)) promises to benefit ~90% of CF subjects, including individuals homozygous for F508del-CFTR and those with F508del-CFTR and a minimal-function mutation [2,3]. However, no therapeutic modalities are currently available for ~10% of CF subjects with rare, or hard-to-treat mutations.

**Fig. 4:**
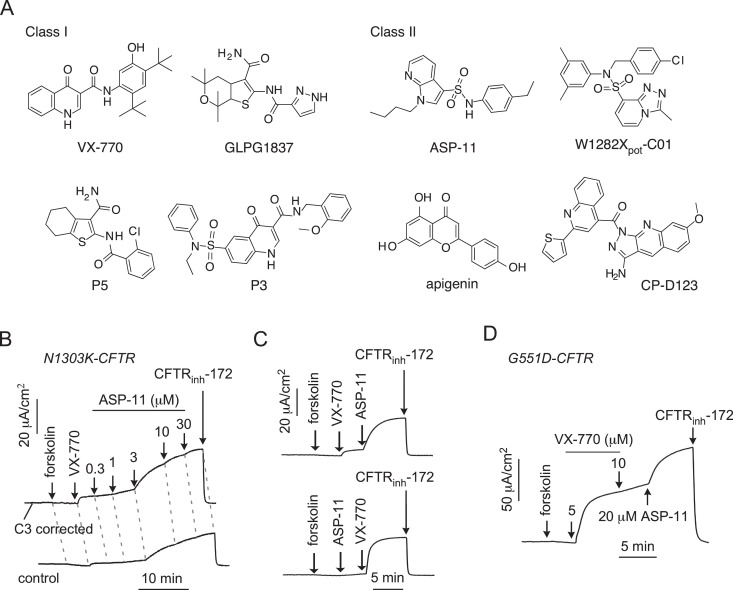
Structure and activity of potentiators and co-potentiators. (A) Chemical structures of the class I potentiators ivacaftor (VX-770), GLPG1837, P3 and P5 (left) and the class II potentiators ASP-11, W1282X_pot_-C01, CP-D123 and apigenin (right). (B–D) Representative recordings of short-circuit current from FRT epithelia expressing N1303K-CFTR (B and C) and G551D-CFTR (D) demonstrate the enhancement of CFTR-mediated Cl^−^ currents by ivacaftor and ASP-11 when used together. CFTR-mediated Cl^−^ currents were recorded using the experimental conditions described in Phuan et al. [74] and in B, the magnitude of N1303K-CFTR Cl^−^ current was augmented by pretreating epithelia with the CFTR corrector C3 (VRT-325 [84]; 3 μM for 24 h at 37 °C). Figure adapted from Phuan et al. [74] under the Creative Commons Attribution License.

Motivated to develop novel therapeutic paradigms for CF mutations that are currently not served by CFTR modulators, high-throughput screening (HTS) was undertaken with CFTR_1281_ (the truncated polypeptide generated by the W1282X-CFTR mutation). Although arylsulfonyl-piperazine and spiro-piperidine-quinazolinone corrector scaffolds with ~5-fold greater efficacy than tezacaftor were identified, efforts to discover new potentiator scaffolds encountered an apparent potentiation ceiling for a single compound [73]. However, when ivacaftor was combined with new potentiators discovered by HTS, including arylsulfonamide-pyrrolopyridines (e.g. ASP-11; Fig. 4), CFTR_1281_ channel activity was enhanced by ~8-fold relative to ivacaftor alone, essentially restoring wild-type-like channel activity [73]. Strikingly, similar synergistic enhancement of channel activity was observed with the ivacaftor / ASP-11 potentiator combination (termed co-potentiation) in Fischer rat thyroid (FRT) cells expressing N1303K-CFTR (Fig. 4) [74]. Consistent with previous studies of CFTR modulators in primary epithelial cells (e.g. [75]), some variability in the response to co-potentiation was observed using epithelial cells endogenously expressing W1282X-CFTR possibly due to protective cellular processes including nonsense-mediated decay and ERAD [73,76,77]. However, co-potentiation with ivacaftor / ASP-11 restored therapeutically relevant levels of channel activity to N1303K-CFTR homozygous primary nasal epithelial cells (i.e. the magnitude of the potentiated short-circuit current was equivalent to that achieved in CF airway epithelial cells (genotype F508del/F508del) after correction with lumacaftor and potentiation by ivacaftor) [74,76]. Using HTS, Phuan et al. [76] identified additional chemical scaffolds (piperidinepyridoindoles, phenylazepines, tetrahydroquinolines and pyrazoloquinolines) that potently co-potentiate with ivacaftor the channel activity of CFTR_1281_ in FRT cells and N1303K-CFTR in primary nasal epithelial cell cultures.

To explain co-potentiation mechanism of action, Phuan et al. [76] grouped CFTR potentiators into two classes. Class I potentiators include ivacaftor and GLPG1837, which interact with a common binding site at the MSD-lipid interface [62,63] as well as P3 (SF-03 ([78]) and P5 (ΔF508_act_-02 [79]) [76] (Fig. 4). Class II potentiators include ASP-11, the flavonoid apigenin and newly identified co-potentiator scaffolds [76] (Fig. 4). Although the binding site of class II potentiators remains to be identified, *in silico* docking studies using the flavonoid genistein suggest that it might be located at the NBD1:NBD2 dimer interface [80]. Phuan et al. [76] demonstrated that co-potentiation occurs when two compounds from different classes are used; when both compounds are from the same class there is no enhancement of CFTR activity. Intriguingly, Phuan et al. [76] found that some CF mutations (e.g. G551D, W1282X and N1303K) are co-potentiation responsive, but other CF mutations (e.g. R334W, A561E and M1101K) were unresponsive. Using a similar strategy, Veit et al. [81] demonstrated that ivacaftor and apigenin potentiate other CF mutations including R352Q, S549N and S1251N. Taken together, these studies identify a novel therapeutic paradigm, and de-risk modulator development for rare CF mutations.

## 6 Future directions

We foresee a prominent role for mechanistic studies to understand CFTR folding, function and pharmacology. We anticipate the application of multi-disciplinary methods to innovative model systems and the use of machine learning to provide insightful analysis of data. Already, Wang and Balch [82] have employed machine learning to investigate genotype-phenotype-CFTR structure relationships.

## 7 Conclusions

The important data that have emerged from clinical trials of triple combination therapies [2,3] suggest that CFTR modulators have wide utility in the treatment of CF. However, CFTR modulators are not available for all CF mutations and modulators with greater efficacy are required to prevent disease progression. A bottleneck in the development of effective CFTR modulators has been a lack of insight into how CF mutations cause CFTR misfolding. Encouragingly, new insights into CFTR structure combined with innovative single-molecule studies using FRET are proving a powerful combination to elucidate the molecular basis of CFTR misfolding and the mechanism of action of small molecules that repair CFTR structure. These approaches are complemented by high-resolution single-channel recording, which provides unique insights into CFTR behaviour, illuminating the action of CF mutations and CFTR modulators. Finally, just as combinations of CFTR correctors have proved decisive in restoring the plasma membrane expression and stability of mutant CFTR [83], the identification of potent efficacious potentiator combinations [73,74,76,81] heralds a strategy to restore wild-type levels of activity to CF mutants. Translation of these potentiator combinations to the clinic should now be a priority.

## Author contributions

All authors drafted the review manuscript or revised it critically for important intellectual content. All authors approved the final version of the review manuscript.

SJ Bose, G Krainer, DRS Ng, M Schenkel, H Shishido and JS Yoon are co-first authors of this work.

PM Haggie, M Schlierf, DN Sheppard and WR Skach are co-last authors of this work.

## Declaration of Competing Interest

HS, JSY and WRS are employees of the Cystic Fibrosis Foundation. DNS is the recipient of a Vertex Innovation Award from Vertex Pharmaceuticals (Europe) Ltd. All the other authors have no conflicts of interest to declare.
